# California's “Bridge to Reform”: Identifying Challenges and Defining Strategies for Providers and Policymakers Implementing the Affordable Care Act in Low-Income HIV/AIDS Care and Treatment Settings

**DOI:** 10.1371/journal.pone.0090306

**Published:** 2014-03-05

**Authors:** Patrick T. Hazelton, Wayne T. Steward, Shane P. Collins, Stuart Gaffney, Stephen F. Morin, Emily A. Arnold

**Affiliations:** Center for AIDS Prevention Studies, Department of Medicine, University of California San Francisco, San Francisco, California, United States of America; Alberta Provincial Laboratory for Public Health/University of Alberta, Canada

## Abstract

**Background:**

In preparation for full Affordable Care Act implementation, California has instituted two healthcare initiatives that provide comprehensive coverage for previously uninsured or underinsured individuals. For many people living with HIV, this has required transition either from the HIV-specific coverage of the Ryan White program to the more comprehensive coverage provided by the county-run Low-Income Health Programs or from Medicaid fee-for-service to Medicaid managed care. Patient advocates have expressed concern that these transitions may present implementation challenges that will need to be addressed if ambitious HIV prevention and treatment goals are to be achieved.

**Methods:**

30 semi-structured, in-depth interviews were conducted between October, 2012, and February, 2013, with policymakers and providers in 10 urban, suburban, and rural California counties. Interview topics included: continuity of patient care, capacity to handle payer source transitions, and preparations for healthcare reform implementation. Study team members reviewed interview transcripts to produce emergent themes, develop a codebook, build inter-rater reliability, and conduct analyses.

**Results:**

Respondents supported the goals of the ACA, but reported clinic and policy-level challenges to maintaining patient continuity of care during the payer source transitions. They also identified strategies for addressing these challenges. Areas of focus included: gaps in communication to reach patients and develop partnerships between providers and policymakers, perceived inadequacy in new provider networks for delivering quality HIV care, the potential for clinics to become financially insolvent due to lower reimbursement rates, and increased administrative burdens for clinic staff and patients.

**Conclusions:**

California's new healthcare initiatives represent ambitious attempts to expand and improve health coverage for low-income individuals. The state's challenges in maintaining quality care and treatment for people living with HIV experiencing these transitions demonstrate the importance of setting effective policies in anticipation of full ACA implementation in 2014.

## Introduction

### Comprehensive health coverage access under the affordable care act

The United States healthcare system will undergo significant changes when major provisions of the Patient Protection and Affordable Care Act (ACA) take effect in January, 2014. Most significantly, access to healthcare coverage will improve as insurance exchanges open across the country and eligibility for Medicaid programs expands in a subset of states. Lower-income individuals in most states will have greater access to comprehensive health coverage, including ACA-mandated coverage for prescription drugs, emergency care and inpatient care, and preventive health tests and screenings. Pre-existing condition exclusions will be eliminated, allowing people living with chronic conditions improved access to affordable health insurance.

These impending changes will result in a significant number of transitions in who pays for patient health coverage. For most conditions, the primary transition will be a shift from patients having no insurance (i.e., self pay or reliance on charity) to a form of coverage (expanded Medicaid or private insurance plan). But for men and women living with HIV/AIDS, the major transition will instead be from an existing HIV-specific payer source to a new comprehensive health payer source.

Because HIV is a transmissible disease with important public health implications, both federal and state governments have moved since the early days of the epidemic to respond with treatment and prevention programs. Currently, access to HIV care is facilitated by the Ryan White HIV/AIDS Program [1], which acts as a “payer of last resort” to assist people living with HIV in the United States in obtaining medical services and treatment for the disease. Ryan White cannot be used to deliver services that patients are eligible to receive under health coverage programs such as Medicaid and private insurance, which will become accessible to a majority of Ryan White patients in 2014. Ryan White itself was up for re-authorization in 2013, and the future role of the program in providing HIV care and treatment services is currently being defined [2].

Research demonstrates the importance of uninterrupted health insurance to achieving desirable HIV health outcomes at affordable cost [3]. Successful HIV care and treatment requires high rates of patient adherence to an ongoing regimen, often within the context of addressing co-occurring health conditions, and there is evidence linking the integrated service delivery of Ryan White clinics to increased patient retention in complex care and treatment regimens [4], [5]. As a result, the transition of people living with HIV to comprehensive health coverage under the ACA will require careful attention to maintaining patient continuity of care and minimizing treatment disruptions.

### California's “Bridge to Reform”

As part of preparations for upcoming changes under the ACA, the state of California received a Section 1115 Medicaid Waiver from the U.S. Department of Health and Human Services (HHS) in 2010 to implement a “Bridge to Reform” demonstration project [6], [7], [8] for the years 2011–2013. The waiver provided money and increased administrative flexibility to implement programs to improve health services funding and delivery, with the expectation that such programs would inform larger ACA implementation, particularly around the Medicaid expansion. California proposed to meet the goals of the waiver with additional investments in public safety-net hospitals and with the rollout of two health coverage expansion initiatives [6].

First, the state mandated that many individuals who are covered under California's current Medicaid program (known as Medi-Cal) transition from a fee-for-service (FFS) model to a managed care model. Since 2011, these patients have been assigned to a single managed care organization, typically a county run health plan, an independent practice association, or an integrated healthcare delivery system (e.g., Kaiser Permanente). The managed care organization assumes the financial risk for the patients' care and, in return, the patient is expected to obtain all care, excepting emergencies, from the organization. Effectively, the model operates much like an HMO option in the private insurance market.

Second, the state chose to create shared-cost, county-run Low-Income Health Programs (LIHPs). These programs are intended to serve as a bridge to the 2014 Medicaid expansion. Each county's LIHP is operated independently of the existing Medi-Cal program, with its own provider network, eligibility criteria, benefits plan, and rules for obtaining care. Each county had the option, but was not required, to set up a LIHP. Starting in July, 2011, California counties began enrolling clients into the LIHPs and moving Medicaid patients from FFS to managed care plans [9]. Each county set different LIHP income eligibility requirements based upon budgetary constraints, with a range between 25% and 200% of federal poverty level (FPL) [10]. Not all California counties chose to create a LIHP, yet all major urban counties (including Los Angeles, San Francisco, San Diego, and Sacramento) had implemented programs by October, 2012 [11], [12], [13], [14].

Both the Medi-Cal managed care and LIHP transitions have been of substantial relevance to low-income people living with HIV (Table 1). The Medi-Cal changes were instituted for all “seniors and persons with disabilities,” including those with HIV. Likewise, the Health Resources and Services Administration (HRSA), which administers the Ryan White HIV/AIDS Program, and the Centers for Medicare & Medicaid Services (CMS) issued guidance in August, 2011, requiring that Ryan White clients in California be enrolled in the LIHPs if eligible [15]. The HRSA and CMS guidance arrived late in the LIHP planning process, with many counties having already made projections for LIHP enrollment assuming that Ryan White would continue to cover HIV patient care and treatment services. In order to comply with the guidance, several counties had to reduce the income threshold for LIHP eligibility, thereby reducing the total number of individuals eligible for coverage.

**Table 1:** Primary Payer Sources for HIV Care and Treatment in California under “Bridge to Reform” (2011–2013).

Payer Source	Key Dates	Eligibility	Services Covered	Provider Networks	Transition challenges
Ryan White/AIDS Drug Assistance Program (ADAP)	In August 2011, federal agencies determine California counties must transition Ryan White/ADAP patients to the LIHPs if eligible.	Uninsured or underinsured people living with HIV/AIDS. Income threshold for ADAP is $50,000 for one person/year. As “payer of last resort,” can only pay for services not covered by other payer sources	HIV-related services, including HIV medical care, medications, medical case management, mental health care, and housing/food services, and nutrition/counseling	All HIV clinics, pharmacies, and service agencies contracted to receive Ryan White funding	Federal guidance not always clear on when Ryan White could continue to pay for services (e.g. medical case management) not covered by the LIHPs and Medi-Cal managed care plans
Low-Income Health Programs (LIHPs)	Created by California's Medicaid 1115 Waiver, approved by federal authorities in 2010. Counties begin enrolling patients in July 2011.	Income requirements vary by county, ranging between 25% and 200% of the federal poverty level (FPL). Not all counties create LIHPs, but all the largest urban counties do.	Comprehensive medical care, including HIV medical care and medications, specialty referrals, and emergency and urgent care. May include, but not required to include, Ryan White-supported services like medical case management.	Includes providers contracted by the county LIHP. Includes some, but not all providers and pharmacies receiving Ryan White funding	Challenges in transitioning patients from providers and pharmacies contracted with Ryan White/ADAP, but not with the LIHPs. Some patients required to transition to providers far from their residence. Not always clear if Ryan White could continue to pay for services not covered by the LIHPs.
Medi-Cal Fee-for-Service (FFS)	As a component of California's Medicaid 1115 Waiver, all “seniors and persons with disabilities” required in 2011 to transition from Medi-Cal FFS to managed care plans.	For most people living with HIV/AIDS, requires permanent disability designation and personal asset threshold ($2000 for one person). Effective in 2011, special exemption from requirement to transition to Medi-Cal managed care necessary for clients to stay enrolled in Medi-Cal FFS.	Comprehensive medical care, including HIV medical care and medications, specialty referrals, and emergency and urgent care. May include, but not required to include, Ryan White-supported services like medical case management	Includes all providers contracted with the state to receive Medi-Cal reimbursement. Includes many, but not all providers and pharmacies receiving Ryan White funding	Often not clear which, if any, patients qualify for special exemption from requirement to transition to Medi-Cal managed care, which may lead to patients being required to switch providers and pharmacies.
Medi-Cal Managed Care	As a component of California's Medicaid 1115 Waiver, all “seniors and persons with disabilities” required in 2011 to transition from Medi-Cal FFS to managed care plans.	For most people living with HIV/AIDS, requires permanent disability designation and personal asset threshold ($2000 for one person)	Comprehensive medical care, including HIV medical care and medications, specialty referrals, and emergency and urgent care. May include, but not required to include, Ryan White-supported services like medical case management	Includes all providers contracted with Medi-Cal managed care plans. Includes some, but not all providers and pharmacies receiving Ryan White funding	Challenges in transitioning patients from providers contracted with Medi-Cal FFS, but not with Medi-Cal managed care. Some patients assigned to new providers who could or would not care for them. Medical exemption and continuity of care requests for patients to stay in Medi-Cal FFS often denied.

A significant number of people living with HIV/AIDS are affected by these transitions. As of June 2012, the Ryan White-funded AIDS Drug Assistance Program (ADAP) served 26,253 clients in California, of whom 71% had incomes at or below 200% of the federal poverty line (FPL), the income threshold for the most generous county-run LIHPs[16]. Nationwide, as of fiscal year 2011, ADAP served 230,932 clients, of whom 59% had incomes at or below 138% of the FPL, the effective income threshold for qualifying for new state Medicaid programs in 2014 [16]. Likewise, it is estimated that as of 2007, 47% of all people living with HIV in care nationwide were enrolled in state Medicaid programs, indicating that a substantial percentage of people living with HIV in California are affected by the transition from Medi-Cal FFS to Medi-Cal managed care plans in 2011–2013 [17].

Unlike Ryan White, the LIHPs and Medi-Cal managed care plans do not specifically focus on HIV care and treatment. Consequently, advocates have expressed concern that the new payer sources would not pay for services traditionally covered by Ryan White [3]. These services include medical case management, pharmacies that provide adherence support (e.g. providing patients with dose packs for complicated daily medication regimens), and assistance in receiving housing and transit benefits. As comprehensive health coverage programs without an HIV focus, the LIHPs and Medi-Cal managed care plans are not likely to reimburse for many of these services. While these programs all include access to HIV specialists, they are not likely to include all HIV specialists in a given geographical area, thereby requiring some patients to switch providers. Likewise, while the LIHPs and Medi-Cal managed care plans are required to provide prescription drug coverage, these programs are likely to restrict patients to a far more limited network of pharmacies than those contracted with ADAP. The county LIHPs have tended to restrict prescription drug benefits to pharmacies affiliated with safety-net hospitals and “medical homes” contracted to provide services to low-income individuals, including pharmacies eligible for HRSA's 340 b prescription drug discount program [18], [19], [20]. This “selective contracting” of pharmacies has the benefit of reducing prescription drug costs incurred by public health plans like the LIHPs [21]. However, potential challenges include the loss of HIV specialty pharmacy services (e.g. medication adherence counseling) and the need to travel greater distances to access eligible pharmacies. Across counties, the LIHPs varied in the degree to which they restricted their pharmacy networks, with some continuing to include HIV specialty pharmacies. As “payer of last resort,” Ryan White/ADAP is prohibited from paying for medications for patients enrolled in the LIHPs and Medi-Cal managed care plans, thereby requiring many patients to switch to a pharmacy in one of the new payer source networks.

HIV patient advocates expressed concern that these payer source transitions would lead to disruptions in care, particularly reductions in antiretroviral therapy (ART) coverage [10], [22]. The worry arose from HIV patients' specific—and unusual—relationship to health reform efforts. Unlike other conditions for which individuals previously could have found themselves without a source to pay for care, low-income men and women already had access to HIV care through the Ryan White Program. Ryan White-funded clinics have incorporated the key tenets of patient engagement, provider teams, and coordination with outside agencies and systems that characterize “patient-centered medical homes,” which the Institute of Medicine has spotlighted as a promising model for chronic disease management [23], [24]. Thus, the significant concern for patient advocates was ensuring that the transitions between Ryan White and new, ACA-related payer sources did not result in people falling out of care [3]. Such an outcome runs counter to the goals of the National HIV/AIDS Strategy [25] and would have significant consequences for individual patients and the US epidemic as a whole.

### Comparison of “Bridge to Reform” and full ACA implementation

California's “Bridge to Reform” programs have important differences with health coverage programs that will take effect with full ACA implementation in 2014. For instance, California's “Bridge to Reform” is a Medicaid waiver, which limits its effects to patients who are eligible for the state's traditional Medi-Cal program, as well as to those who are newly eligible for the county-run LIHPs (which expand coverage to some or all patients in each participating county who will be eligible for Medi-Cal in 2014). In contrast, the new health coverage programs in 2014 include a full expansion of Medi-Cal to all legal residents with income under 133% of the FPL, as well as the creation of a new health insurance exchange named Covered California, with federal subsidies available to patients with incomes between 133% and 400% of the FPL. As a result, many more patients in California will become eligible for comprehensive health coverage under full ACA implementation than under “Bridge to Reform.” Nationwide, it is estimated that an additional 124,000 people living with HIV could gain new health coverage under the ACA in 2014 [26]. Additionally, one of the key “Bridge to Reform” programs – the LIHPs – will end once the ACA is fully implemented in 2014. Finally, most other states did not include a Medicaid waiver program, and in some cases have a different underlying structure of health care delivery, and this may lead to differences in how full ACA implementation rolls out across the country.

At the same time, “Bridge to Reform” is anticipated to have many important similarities with new health coverage programs under full ACA implementation. For instance, as more patients across the state become eligible for Medi-Cal in 2014, there will be transitions away from HIV-specific Ryan White coverage similar to the transitions experienced by patients moving into the LIHPs between 2011 and 2013. Likewise, as Medi-Cal managed care and Covered California plans cover more patients in 2014, it is anticipated that HIV clinics, pharmacies, and support services agencies will increasingly need to negotiate to contract with these plans as they did with Medi-Cal managed care plans in 2011–2013.

### Purpose of this analysis

California's “Bridge to Reform” programs constitute a naturally occurring experiment. In order to assess the potential impact of the transition in payer sources, we conducted semi-structured, in-depth interviews to better understand the perspectives of HIV providers and policymakers during the implementation of these two state initiatives. We specifically sought to characterize the challenges of transitioning low-income HIV patients to new payer sources and to identify potential strategies for minimizing problems and maintaining patient continuity of care. We did not include patient interviews in this analysis as we anticipated challenges in gathering a sufficient sample of patients familiar with details of the health coverage transitions. The findings that we describe here are intended to provide data that highlight the challenges that California faced during its recent efforts to transition HIV patients to new payer sources, to draw potential lessons from this experience that speak to the kinds of challenges that may arise during similar transition efforts (e.g., as ACA reforms are rolled out on a wider scale), and to identify potential strategies for mitigating challenges during future transition efforts.

## Methods

### Ethics statement

The University of California San Francisco's (UCSF) Committee on Human Research reviewed and approved all study protocols, including the use of verbal informed consent. Because the primary risk associated with this research was privacy, we explicitly sought and received approval from the Institutional Review Board to use verbal consent procedures instead of written consent with our participants. The verbal consent procedures involved having a research team member provide verbal informed consent, wait to receive a verbal response from the participant assenting to participation, and then sign an informed consent form on behalf of the participant.

### Data collection and analysis

A multi-disciplinary team of researchers conducted 30 in-depth, semi-structured interviews between October, 2012, and February, 2013, with respondents in 10 urban, suburban, and rural California counties. Participants consisted of public health policymakers, medical providers, pharmacists, and service providers, which includes social workers, case managers, and benefits counselors (Table 2). Participants were selected in consultation with community collaborators who had been connected to both clinics and local county policymakers across the state as the Medicaid managed care and LIHP transitions took place. Participants were contacted via email or phone and invited to take part in the study.

**Table 2:** Sample Characteristics.

Variable	Category	Value
Participant role (*n*, %)	Clinic/Agency administrator	7 (23%)
	Pharmacist	3 (10%)
	Medical Provider	2 (7%)
	Policy maker*	8 (27%)
	Service provider**	10 (33%)
Region	Northern California	14 (47%)
	Southern California	16 (53%)
County	Alameda	4 (13%)
	Butte, Colusa, Glenn, Shasta, Sutter, Tehama, Trinity, and Yuba***	1 (3%)
	Contra Costa	1 (3%)
	Los Angeles	7 (24%)
	Orange	2 (7%)
	Riverside	2 (7%)
	Sacramento	1 (3%)
	San Bernardino	1 (3%)
	San Diego	4 (14%)
	San Francisco	6 (20.0%)
	Sonoma	1 (3%)
Setting	Rural/Suburban	9 (30.0%)
	Urban	21 (70%)

*Includes County level LIHP directors, ADAP directors, and HIV Program Managers. Some of these individuals also were practicing HIV physicians.

**Includes social workers, case managers, and benefits counselors.

***One participant served all eight of these rural counties in Northern California.

After completing verbal consent, a member of the research team interviewed each participant for 45–90 minutes in a private office setting or by phone. Interview topics included: continuity of patient care, integration of wrap-around support services, capacity to handle payer source transitions, administrative burden, and preparations for healthcare reform implementation in 2014. Emergent themes in the data also informed the selection of additional interview participants, and related topics were then incorporated into the semi-structured interview guides. Sampling continued until theoretical saturation was achieved, signifying no new themes or data emerged in the interviews. Interviews were recorded and subsequently transcribed. Any identifying information was redacted from the written documents.

Analyses were performed using the tenets of grounded theory, one particular approach to qualitative data collection and analysis, and began while the team was still in the field collecting data [27]. During analysis meetings, study team members reviewed interview transcripts to produce emergent themes, which were used in conjunction with the interview guide to develop a preliminary codebook. From this preliminary codebook, code names and definitions evolved to match emerging data during iterative analyses of the interviews by project investigators. Through reading and coding four common transcripts, coder agreement reached 90%, at which point the raters completed final coding of the dataset. Memos were also written to build theory about coding decisions and cross case analysis. Four research team members met regularly to build coding consensus, to become familiar with participant narratives, to contextualize discrepancies, and to make coding and cross-case analysis decisions of newly uncovered themes. Codes were applied to the transcripts using the Dedoose software platform, which allowed investigators to perform searches and compare findings across cases. Quotes selected for inclusion reflect the experiences and views of participants, and were chosen based on their relevance to the study goal.

## Results

Five common themes emerged around the challenges and opportunities faced by providers, patients, and policymakers in maintaining continuity of HIV care during the Medi-Cal managed care and LIHP transitions: (1) positive perceptions of the new comprehensive health coverage offered under the Affordable Care Act; (2) perceived inadequacy in LIHP and Medi-Cal managed care provider networks for delivering quality HIV speciality care; (3) the potential for clinics to become financially insolvent as a greater number of patients are moved to payer sources like Medi-Cal with low reimbursement rates; (4) a need for more effective communication strategies to reach patients and establish partnerships between providers and policymakers; and (5) increased and confusing administrative burdens. These themes are discussed in more detail below.

### Positive perceptions of new ACA-related comprehensive health coverage

Participants generally expressed positive views of the comprehensive health coverage to be offered through the ACA. At the same time, participants characterized implementation challenges associated with the current payer source transitions as ones that would need to be addressed in order to minimize difficulties with larger ACA implementation in 2014. As stated by one clinic administrator director in San Francisco County:

*LIHP is actually – or SF Path [the name of the local San Francisco LIHP] is actually a great opportunity. It's a better, more comprehensive insurance package for people that don't currently have insurance. So we support the concept.*

Several providers described how the LIHPs and Medicaid managed care would allow patients access to health coverage that was previously unavailable when Ryan White was their sole health coverage payer source. A service provider in Los Angeles County explained the benefits of the new payer source coverage in detail:

*At least under managed care, there are services that are going to be provided under managed care that are going to benefit Ryan White patients, and in Ryan White, inpatient isn't covered, ambulance isn't covered, and that's a challenge for our patients right now, where they come to us and they say, look, I had to use the – an ambulance, and I called 911, who can help me pay this – and there's no funding to cover an ambulance bill. So those are the things that come up, and I think – from a broader perspective, that if we're looking at hospitalization and in-patient care, of course that's going to be better under managed care.*

This provider's description of services covered by the new payer sources that could not be reimbursed under Ryan White, such as hospitalization and inpatient care, reflects a common perspective among participants that the new health coverage options under the ACA were likely to be less HIV-specific and more inclusive of key health care services needed by their patients.

### Perceived inadequacy in the provider networks to deliver quality HIV care

Even as many participants described the benefits of new comprehensive services offered by ACA-related payer sources, numerous providers and policymakers expressed concerns about the more limited HIV-specific resources within the LIHP and Medi-Cal managed care provider networks. One clinic administrator in Los Angeles County thought that HIV was inadequately considered when the county-run LIHP network of providers and pharmacies was created: *“This one felt like they – someone else built the networks and never really coordinated the efforts that were going together.”* This participant went on to describe how the Medi-Cal managed care transition initially caused some clinic patients to be switched to primary care providers without HIV expertise, raising concerns about the quality and continuity of their HIV care:

*The doctor's not necessarily going to be an HIV specialist, right? So the doctor's not going to be familiar with whatever signs, symptoms, issues, illnesses, medications the client's on, yet this is supposed to be the new primary care physician. So without a specialty clinic which is really designed to take care of the client, keep them healthy, reduce the viral load, increase the T-cell count, and reduce or eliminate the transmission of the virus, now we have a client who may go without treatment and ends up getting turned away and is frustrated.*

To address these and other network adequacy concerns, a Northern California policymaker suggested that HIV clinics work together to ensure they are included in managed care plans: *“So the clinics need to make sure that they stand up, be counted, and start getting their act together to be able to be part of a managed care network.”* Yet, as expressed by a service provider in Los Angeles County, the process of contracting with individual Medi-Cal managed care plans is complex and poses particular challenges for HIV-specialty providers and clinics:

*So one health plan will say, well, we don't have – we don't identify providers as HIV specialists in our provider directory, and we don't have a way to link patients with HIV to an HIV provider because, from our perspective, we don't look at the patient's diagnosis when linking a patient to a provider. And then you'll find another health plan that will say, okay, we have an area where we identify providers as HIV specialists, but then again, once your provider is identified as a primary-care provider, you have to see all patients that are then assigned to that provider, and they're not all going to be with an HIV diagnosis.*

Providers and policymakers also described the particularly acute challenges caused by HIV-specialty pharmacies being left out of the LIHP and Medi-Cal managed care networks. A pharmacist representing an HIV specialty pharmacy in a northern urban California county explained that the local county LIHP did not include HIV specialty pharmacies like her own, creating concerns that her patients would be unable to meet their daily medication needs at a regular pharmacy:

*HIV drugs are so expensive. Most regular pharmacies do not keep them in stock. So if you have a patient walking in, that needs to start treatment, or is out of meds and needs them that day, most of the time you would not have them unless you're a specialty pharmacy.*

Several providers discussed concerns that the new payer sources either included or would include a limited scope of benefits with insufficient coverage for case management services, which they perceived as critical to assisting vulnerable patients with limited capacity to handle new health plan requirements. One clinic administrator working in several rural northern counties described concerns about anticipated managed care implementation in 2014:

*So if [a new HMO] moved in here today and all of our clients all suddenly had to go to [that HMO], where's the expertise for any case management? It's gone! Unless [the HMO] were to agree to contract with us to provide it – which – in a perfect world, I guess that's what would happen – plus, [the HMO] would contract with our local HIV providers.*

Likewise, a service provider in Los Angeles County discussed the need for Ryan White-financed clinics and AIDS service organizations to plan for anticipated challenges as new managed care provider networks with a limited scope of benefits are implemented:

*I think that you should be able to make sure that you have the systems in place for eligibility, make sure that the pharmacy piece is in place – look at the critical components that are currently offered under the Ryan White program, and then how are those services going to continue in a managed care environment.*

As this provider states, one potential strategy to address network adequacy entails examining the entire patient system of care, ensuring that services covered under Ryan White continue to be covered when patients transition to managed care plans. A clinic administrator in Sacramento County described how his clinic was able to maintain medical services for patients by developing a protocol for enrolling his clinic's medical providers as primary-care providers in managed care plans:

*Usually – it's filling out the forms, faxing it or in some way delivering it to the health plan, and then getting them to review it, and then if there are any questions, they come back. Sometimes there's some dialog, and then getting entered into their electronic system in order so it's recognized as a primary-care provider.*

This provider's experience in enrolling his clinic's medical providers as primary-care providers in the new managed care plans illustrates a recurrent trend in the data. Over time, HIV clinics, pharmacies, and support agencies developed strategies to contract with managed care plans and LIHPs, thereby improving the network of HIV services available to patients transitioning into the new plans.

### Potential for financial insolvency as more patients are moved to payer sources with low reimbursement rates

The issue of financial solvency, and the substantial investments needed to obtain certifications that would allow for enhanced reimbursement rates such as becoming a federally qualified health center (FQHC) [28] or a patient-centered medical home (PCMH) [29], weighed on providers and clinic directors. These concerns arose from the fact that upcoming ACA transitions will result in many patients moving from a relatively more generous payer source (i.e., Ryan White Program) to a relatively less generous payer source (i.e., Medi-Cal). FQHC's are eligible for enhanced reimbursement rates under Medi-Cal, and there are grant monies available for clinics to restructure as FQHC's or PCMH's in anticipation of ACA implementation. Indeed, some private insurance companies offer PCMH's increased reimbursement rates, which for HIV providers in mixed payer practices, made it an attractive model of care. With ongoing cuts to Medi-Cal reimbursement rates in California, which further exacerbate the state's historically low Medicaid rates[30], there is already increasing reliance on using Ryan White funds to support comprehensive services and to supplement medical costs not absorbed by Medi-Cal. But as the proportion of patients covered by Medi-Cal increases and the proportion covered by Ryan White decreases, the strategy will become less viable. This trend, coupled with constrained clinical revenue flows, has resulted in fears that clinics will not be able to increase capacity and remain afloat under the ACA. As stated by one clinic administrator in San Francisco County:

*The reimbursement rates are a big concern for program sustainability. We heard just this morning that the courts overturned the ruling that [had called for Medi-Cal reimbursement levels to remain the same]… the legislature had approved a ten-percent decrease in provider fees for Medi-Cal… So it looks like we'll be seeing cuts in Medi-Cal reimbursement…financial sustainability is our main issue for HIV services.*

This provider went on to emphasize the importance of Ryan White Program funding in paying for essential comprehensive HIV care and treatment services provided by the clinic:

*[Ryan White covers] case management, pretty much any of the supportive services, [and] actually, the cost level of the medical services as well. So Medi-Cal provides a certain reimbursement, and Ryan White fills in those gaps of anything that isn't covered by Medi-Cal.*

This provider indicated that Ryan White funds would be needed to maintain the level of comprehensive services at the clinic, as well as its financial viability.

One clinic administrator in Los Angeles County agreed, and emphasized the financial challenges of moving from Ryan White as a payer source to new payer sources. Ultimately, the inability to adequately bill for comprehensive services led to cuts, negatively impacting patients:

*Well, the reimbursement rate [from Medi-Cal] is significantly different, and I think the level of care and the continuity of care we've been able to provide under the Ryan White funding, I think is going to be substantially different, because everything's moving…to be at a rate that's significantly less.*

Participants expressed concern that under the ACA, clinics would need to make investments in order to achieve the necessary standards to become FQHCs and qualify for higher reimbursements from Medi-Cal. As one health agency administrator in Los Angeles County observed, *“I think with the move to managed care and with health reform, clinics are going to have to become federally-qualified health centers if they're going to survive.”* Yet with reduced sources of revenue, it was not clear where investments to make that transition and to maintain comprehensive levels of care would come from. As stated by a clinic administrator in Sacramento County:

*As more of a businessperson, there's two aspects that I think are really key, is that without adequate reimbursement, the community clinic system cannot expand capacity. I'm not talking about one-time grants, I'm talking about ongoing revenue streams for providing care. It can't be done. It'll end up in a reduction in the breadth of services comprehensive care that is being seen as the way of the future. This patient-centered medical home thing, that's not an inconsequential investment. And so if there's one thing that I'm concerned about, is adequate reimbursement, to be able to support the concepts that are presented in a patient-centered medical home. So that's a concern. The other concern is that providers, specialty providers, that they be willing to take these managed-care patients. And so we have a robust referral network that we can go to that will accept our patients. Those are two really broad areas that I think need to be focused on, or these things aren't going to happen.*

As stated by this clinic administrator, clinics will need to focus on multiple strategies in order to maintain access to care for patients under new ACA programs. These strategies include engaging in pursuing certification as a patient-centered medical home, which as the clinic administrator notes, would require many years of investment in clinic infrastructure and training. Other strategies include advocating that the state Medi-Cal program set sufficient reimbursement rates to attract an adequate specialty referral network for HIV patients.

### Need for new communication and partnership strategies

In general, policymakers and providers felt that the transitions of patients to Medi-Cal managed care networks and LIHPs were rushed and exposed significant challenges in communication between providers and patients, as well as between providers and policymakers. One clinic administrator in San Francisco County described initial challenges communicating with the county public health department regarding the LIHP implementation:

*We were getting communication from [the county public health office] around …LIHP, late 2011, early 2012. And that's when we were supposed to be implementing…I know that other conversations had been happening, but they were very preliminary conversations. They were not official communication tools – PowerPoints and webinars and pamphlets for clients. So I think more community engagement with those communication tools further in advance would be helpful. The community forums to express concerns didn't seem to happen until after they had already released their strategy. So it was kind of like, well, these are our concerns, but you're already going to do what you're going to do.*

In response to the initial gaps, providers and policymakers have adopted new communication strategies and partnerships to assist in both the ongoing transitions and the anticipated ACA transitions in 2014. A Northern California policymaker emphasized the particular importance of creating a communication strategy to reach patients:

*Then at the clinic level, the first thing is, I tell people, you need to know how to reach your patients. So if you have not paid attention to that now, you need to ask them fourteen times, how can we find you, even if you're – who always knows where you are?*

Likewise, a Southern California policymaker described specific strategies to improve communication with patients:

*I think that we have a process in place for disseminating information and communicating with patients. Via the one-on-one's, the FAQ documents, the planning council and provider-client advocacy type of groups, to communicate those changes, and I feel like that communication network has been working.*

A medical provider in Alameda County stated that improved communication with patients could also translate into effective policy action, as patient stories are collected and communicated onwards to policymakers:

*Considering that lens, in a very direct way, trying to squash the hierarchy so the people at the top are as close as possible to the patient experience so they can make decisions that make sense – really making sure you understand the entire chain until the pill gets into someone's mouth.*

Yet, as a clinic administrator in San Francisco County expressed, communication with patients and among providers themselves could be significantly improved to more adequately prepare for anticipated transitions:

*For those of us who know, we know that we're going to start doing enrollment for the Medi-Cal expansion possibly as early as July, but definitely by October. So that's only six months away. And outreach and engagement of those clients who are going to be newly eligible is going to be really important, but I haven't seen any strategy around that. So no, I don't think there is enough cognizance among on-the-ground providers that this is what's happening and that they're ready for it.*

Several policymakers and providers discussed specific strategies for improving communication among providers and policymakers themselves, particularly between HIV-specific stakeholders, such as HIV and STD sections of county departments of public health and non-HIV-specific stakeholders, such as county departments of health care services. A Southern California policymaker described strategies implemented at the county-level to improve this communication:

*Behavioral health's at the table, transitional systems department, our human services systems, program integrity and development, everybody. I mean, everyone's represented. And it's a standing meeting, and even if the directors or their assigned people can't go, someone always attends. So that there's a continued dialogue.*

Like this policymaker, numerous other providers and policymakers described communication processes adopted at the county-level to improve dialogue among key stakeholders for ACA implementation. These processes were frequently described as being set up in response to what felt like rushed transitions of patients into Medi-Cal managed care networks and the LIHPs in 2011–2012. By instituting processes like regular stakeholder calls and meetings, development of FAQs and checklists, and the creation of “healthcare reform task forces,” these providers and policymakers hoped to more effectively communicate information regarding the more widespread transitions anticipated to occur with ACA coverage expansion.

### Increased and confusing administrative burdens

Many providers reported experiencing increased administrative burdens, either for themselves or their patients, as a result of the transitions to new payer sources. Participants described the substantial impact of this administrative burden – primarily in the form of eligibility verification requirements and enrollment processes – on patient continuity of care, in many cases leading patients to experience disruptions in care, including disruption in ART medication adherence. A service provider in San Diego County described an example of this “overwhelming” new burden experienced by patients:

*The information that Medi-Cal gives the patients is very confusing to most patients. We live in a society that – a lot of people don't really go over their benefits. So it's – when someone comes up with – a big packet with five, six booklets, with thousands and thousands of doctors and clinics included in there, for the patient, it's overwhelming to go to look at the information.*

Likewise, a policymaker from Southern California noted how socioeconomic challenges experienced by many HIV patients, such as homelessness, compounded the difficulty in navigating these new administrative requirements:

*When they actually go through the [Medi-Cal and LIHP] application process, it's like, okay, we need you to bring in your vehicle registration, we need you to bring in your medical bills, we need you to bring in – the list of things is very challenging. We have folks who just – they're homeless. They don't have that stuff. They're eligible for the program, but they don't have it and they give them a ten-day period to bring it in, and they don't say, well, yeah, I'm not going to be able to get it in ten days. Would you give me 30 days, or could you extend that time? They don't ask for that. So they soon get a notice of action saying, you didn't comply with the process, and therefore you're denied Medi-Cal or LIHP, and so now they have failed to comply with a process. So it's the complexity of that application process.*

Providers discussed how new administrative requirements were particularly likely to affect continuity of patient care at the pharmacy level, when patients attempted to refill prescriptions but found themselves unable to do so, resulting in days or weeks without ART treatment. A Northern California policymaker explained that this disruption in care occurred for several reasons:

*Mostly what has happened is, just at a very basic level, patients have a prescription that's been written by the provider. They show up at a pharmacy which they believe will be able to fill it and are told that it can't be filled. And that happens for a number of different reasons. Their ADAP [AIDS Drug Assistance Program benefit] has expired and they haven't done what they need to do to renew it. Their ADAP expired and they were supposed to sign up as [LIHP], but haven't completed that process. Or, they are – they have signed up for [LIHP], but they don't understand the difference, and they take the prescription to an ADAP pharmacy that's not a [LIHP] pharmacy.*

In addition to the direct impact on patients, providers reported that patients were indirectly affected by the challenges the providers, themselves, had encountered when navigating the new administrative requirements of the LIHPs and Medi-Cal managed care programs. These providers discussed how support staff members, such as case managers and social workers, were increasingly likely to spend their time providing benefits counseling rather than providing direct social services or adherence counselling to patients. The San Diego County service provider quoted above further explained:

*There's so much paperwork to push out, and dealing with the increased population that comes in all the time with benefits problems, so mostly, my appointments, before, I used to have three or four patients at least for educational needs or reinforcing adherence, treatment follow-ups and things like that. It's almost nonexistent. Now it's, I have problems with my Medi-Cal. I have problems with my prescription plan. I have my ADAP renewal, my Ryan White is cancelled, my LIHP is not pending, my Medi-Cal worker is not giving me this, my – this and this – constantly, constantly, the same thing over and over again.*

Several providers discussed how the heightened administrative burden was causing difficulties in maintaining support staff morale. These providers expressed concerns that support staff like social workers and case managers were now conducting tasks misaligned with their educational training and prior clinical experience. One service provider in Alameda County discussed the considerable direct impact on these providers, in addition to the indirect impact on patients:

*I think one of the most important ways our patients have been affected is indirectly through the amount of time their providers are filling out forms and obsessed with payment issues, rather than directed at providing care. So our social work staff has become consumed – are up in arms – literally, close to people quitting their jobs – serious, serious deformation to the work life of long-term, highly experienced, clinically skilled staff, who are spending their time on the phone, filling out forms, and doing just BS work. All the way up to and including docs, but really, it's fallen most heavily on our case management staff.*

Several providers also discussed how their clinics had implemented specific strategies to alleviate the heightened administrative burden caused by transitions to new payer sources. One approach used by several clinics was to identify private funding, from local foundations or other sources, to hire benefits counselors or additional case managers. One clinic administrator in Riverside County discussed the benefits of this strategy:

*It's now been a year and a half [since implementing the Medi-Cal Managed Care and LIHP transitions], we have increased the number of case managers we have, by three. Our client caseload hasn't increased. So it has just been a much larger burden to navigate these new health plans, because our client population, if they didn't have the case manager, would fall out of care. And I'm making a generalization. But there is – if anyone is more likely to be falling out of care, it's somebody we're serving. And so we have had to find private funds to hire additional case managers, so that caseloads are smaller, and that they can give more time to navigating the system changes.*

Similarly, one clinic administrator in Los Angeles County described the benefits of the county contracting a Medi-Cal eligibility worker to visit the clinic one day a week to assist patients with navigating the Medicaid managed care transition:

*We have the Medi-Cal lady, who does the assisting with the new health care reform for Medi-Cal, so working out the managed care plans with the clients. She comes in one day a week […] and she just assists clients who have questions about – now going from fee-for-service Medi-Cal to this new Medi-Cal managed care, where they have to select a plan, or they're trying to opt out of the plan, so she can kind of assist them in selecting a plan, or whatever it is that they need, she assists them with that, so she's actually here on site one whole day a week.*

Despite these successes, numerous providers talked about budgetary concerns that limited the ability of their clinics to hire new case managers or benefits counselors, including one clinic administrator in San Francisco County: “You know, it would be lovely to be able to hire more people. In our current budget, it's not going to be feasible…” Although these providers expressed a significant need to identify new hiring and training opportunities to alleviate administrative burden, they stressed the challenge of doing this within the financial constraints faced by their clinics.

## Discussion

Our findings demonstrate that many providers and policymakers active in California's “Bridge to Reform” programs hold positive perceptions and concerns about ACA implementation consistent with existing research on views of the ACA within the healthcare field [31]. While providers and policymakers in our study viewed the ACA positively for its potential to expand health coverage to the uninsured, they also identified concerns that patients will face challenges in accessing high-quality HIV care. In particular, our study identifies challenges unique to patients transitioning from the HIV-specific services of the Ryan White Program to the comprehensive coverage provided by new ACA plans. The success of the Ryan White Program in providing high-quality, interdisciplinary models of HIV care is well-documented [32]. However, due to the novelty of the ACA, there currently exists little research on the implications of the ACA in maintaining and expanding high-quality HIV treatment among low-income populations, a deficiency in part addressed by this study.

Our research on the impact of the transition to Medi-Cal managed care and the implementation of LIHPs, both components of California's “Bridge to Reform,” highlighted four perceived policy challenges that are likely also to be present in varying degrees during larger ACA implementation. Each of these challenges has the potential to be addressed with adequate planning, as summarized in Table 3.

**Table 3:** Identified Challenges and Strategies for HIV Care and Treatment in California under “Bridge to Reform” (2011–2013).

Challenges	Strategies
Network adequacy	HIV providers negotiate with managed care plans to contract as primary care providers or HIV specialists. Some LIHPs contract with pharmacies that offer medication adherence services. HIV clinics begin planning and investment to be certified as “Medicaid health homes” eligible for enhanced reimbursement under the ACA.
Financial solvency	Ryan White continues to cover the cost of HIV-related services (e.g. case management) that are not covered by all ACA-related plans. In states not yet expanding Medicaid, Ryan White/ADAP could continue to cover the cost of HIV medications, and potentially be used to purchase or subsidize health coverage through health insurance exchanges.
Communication	Local and state agencies create client FAQs describing healthcare reform details, hold regular calls between HIV-specific and non-HIV-specific health stakeholders, and use patient advocacy groups to disseminate information.
Administrative requirements	Patient advocates, navigators, social workers, and case managers help clients with limited health literacy complete verification requirements. Advocacy groups work with state Medicaid programs to streamline the application process, and allow the same forms to qualify individuals for multiple programs.

### Challenge: network adequacy

Our participants expressed concerns about the sufficiency of HIV expertise within the new managed care provider networks. This worry has particular salience in tough budget climates where a greater emphasis is placed on controlling healthcare costs through the use of tighter managed care rules. Patients assigned to HMO-style programs do not have the freedom to seek specialized care outside of their approved provider network, at least not without specific authorizations that can be cumbersome to obtain. Participants also expressed concern that the new networks would limit coverage of case management services and access to HIV specialty pharmacies, which may negatively affect treatment outcomes.

### Strategies

In our study we found that providers and managed care plans often found a way to work around this problem over time. As providers themselves became acquainted with new managed care plans, many became contracted as primary care providers or HIV specialists within the networks of individual plans. This often required time and careful planning in order to meet plan requirements. This process was made easier by the existence of managed care plans in California that have evolved successful HIV specialty care. Also, some LIHPs contracted with pharmacies that provide HIV specialty services, like home delivery and provision of medication dose packs with pre-packaged doses for patients with complex treatment regimens. In our study, we were unable to determine exactly how many LIHPs contracted with pharmacies offering these services, though it was clear from our interviews that some did while others did not. In addition, the ACA contains incentives to create a “Medicaid health home,” a patient-centered medical care model that provides enhanced reimbursement rates for clinics that achieve successful patient outcomes for costly ailments like HIV [33]. Several participants in our study discussed their efforts to certify their clinics within a patient-centered medical care model. This concept may further improve the ability of HIV specialty providers to operate within a Medicaid managed care model in the coming years. Again, advanced planning and adjustments after implementation will be important considerations at the state level.

### Challenge: financial solvency

Our participants also expressed worry about the overall viability of their HIV specialty clinics as more patients are moved to payer sources with very low reimbursement rates. This trend will be potentially problematic for all providers or clinics that serve low-income patients. But it is a particularly salient worry for HIV speciality clinics that have traditionally been able to use Ryan White funding to create comprehensive services in impoverished, high-risk communities. Such clinics may not be able to survive unless they are able to construct business models that offset low Medicaid reimbursement rates, particularly in states like California with severely low payments. There has been little research specifically on the adequacy of Medicaid reimbursements in the context of HIV care. But studies have shown that *Medicare* rates may not be adequate for covering the full cost of HIV care and treatment services [34], [35]. If this is true, then the situation is likely even more problematic with Medicaid payments, as its reimbursements are lower than Medicare's[36]. Furthermore, California's spending per Medicaid enrollee has historically been the lowest in the nation [30], making the problem of inadequate Medi-Cal reimbursements especially severe. Individual providers may need additional incentives to contract with Medi-Cal managed care plans, as many providers have been unwilling in the past to accept Medicaid as reimbursement [37].

### Strategies

Potential solutions to improve financial solvency proposed by our participants include the use of the Ryan White model for HIV specialty care with modifications to offset some of the current expenses through Medicaid expansion. In states not yet expanding Medicaid, ADAP may be used in some cases to purchase or subsidize health coverage through health exchanges [38]. Thus, in some cases maintaining the system of Ryan White clinics with adaptations to the ACA may be policy options.

### Challenge: communication

Providers and policymakers highlighted the challenges of communication. Quite simply, there is a very large amount of information to be transmitted to a very large number of people, a complexity that will likely become more pronounced during full ACA implementation. The absence of good information flow results in confusions and complications in managing patient care and clinical operations.

### Strategies

Participants identified a variety of strategies to improve communication with patients and among providers and policymakers. These included the development of client FAQs describing healthcare reform details, regular calls and meetings between HIV-specific and non-HIV specific health stakeholders, and the use of patient advocacy groups to disseminate information. The San Francisco HIV Health Care Reform Task Force has created a provider planning document that recommends similar strategies, including identifying key staff both within and outside the clinic to assist patients in understanding the payer source transitions, and addressing the linguistic and cultural diversity of patient populations [39].

### Challenge: administrative burden

Providers and policymakers cited heavy administrative burdens that were confusing and time-consuming for the staff at HIV clinics and for their patients. To the degree that such burdens prove overwhelming or confusing to patients and providers, they place patients at increased risk of experiencing a disruption in HIV care. This problem seems especially likely to occur among individuals with limited healthcare literacy, those with unstable housing (and, by extension, unstable mailing addresses), and/or those who have trouble supplying eligibility documentation.

### Strategies

Our participants discussed several strategies to reduce administrative burden challenges. These include proposing at the state level to streamline the application process for Medicaid and to allow the same forms to qualify individuals for multiple programs. Likewise, participants mentioned the need for increased patient assistance as provided by social workers, case managers or patient navigators. Planning for this increased assistance in advance is important to minimize disruption.

Patients and providers are also anticipated to receive enrollment support from Covered California and private insurance exchanges in other states, which will employ individuals who will provide advice on participating in the expanded Medi-Cal program or in health plans offered through the new exchange [40], [41]. However, making use of these services will likely require a phone call to an unfamiliar and unknown entity (e.g., the insurance exchange). For particularly disenfranchised patient populations, more intensive forms of help will likely be needed. Funding for such positions would be a vital step toward ensuring that the most vulnerable patients do not fall out of high quality HIV care. To that end, Covered California has also awarded contracts to 48 lead organizations to conduct intensive outreach in diverse communities [40].

### Continued importance of the Ryan White program

A major implication of our findings is the continued importance of the Ryan White HIV/AIDS Program, even in an era of expanded access to other forms of coverage. The program was due for re-authorization in 2013, and its future funding levels are uncertain because a large number of its clients are anticipated to gain Medicaid or private health insurance in 2014 [42], [43]. The experience of the state of Massachusetts is instructive here. The state was able to reduce new HIV diagnoses and achieve very high levels of viral suppression during the rollout of its state healthcare reform. But those successes were possible only because it was able to ensure continuity of care through services funded by the Ryan White Program [2], [44].

Continued Ryan White Program funding may provide a lifeline to clinics in disadvantaged settings that could not otherwise create a sustainable funding model, especially given the very low reimbursement rates in many state Medicaid programs. It is possible that such clinics might eventually develop new business models to support their services, such as being certified as a federally qualified health center (FHQC) or patient-centered medical home (PCMH) in order to obtain higher reimbursements [23], [45], [46] from public or private insurance sources. But putting in place the procedures, information systems, and capacities for these certifications takes time and money. The Ryan White Program provides a mechanism for ensuring continued clinic survival while new business models are developed.

### Limitations

Our study has several limitations. First, because there are differences between “Bridge to Reform” and full ACA implementation, the challenges that we identified will not encompass all obstacles likely to occur in 2014. For instance, “Bridge to Reform” is a Medicaid waiver and therefore has not produced the kinds of barriers that patients may face when enrolling in private insurance plans through Covered California. “Bridge to Reform” also does not affect as many patients as those who will enter new programs in 2014, and there is the possibility that HIV clinics, pharmacies, and support agencies will face new challenges in contracting with private health plans in 2014. However, given the many similarities between “Bridge to Reform” programs and those that will take effect in 2014, we anticipate that the four key policy challenges identified in our research will continue to affect the provision of care and treatment in 2014 and beyond. Second, we did not include patient interviews in this analysis, as we anticipated difficulties in identifying a sufficient number of individuals who would be able to describe in detail their experiences with the health coverage transitions. We also did not interview many medical providers, as we found that they were less likely to be aware of the details of the ACA-related transitions than clinic administrators and support staff. It is possible that interviewing medical providers by level of caseload could deepen perspectives on the perceived requirements for HIV specialists in providing high-quality care. Third, our findings are partially a reflection of California's specific contextual factors, which may or may not be similar in other states. For instance, states will differ in whether they choose to expand their Medicaid programs and, among those that do not expand Medicaid, whether they adopt a subsidized private market approach in lieu of the expansion. States also differ in the dynamics that influence non-Ryan White funding streams. California, for example, has particularly low Medi-Cal reimbursement rates. Clinics in states with higher rates may not be as heavily affected by reductions in Ryan White funding. Despite these limits on generalizability, it is important to keep in mind that the purpose of our study is not to measure the exact prevalence of challenges experienced by HIV patients and providers transitioning to new ACA-created payer sources, but rather to understand the landscape of potential challenges that may arise during payer source transitions and to identify the types of strategies that could mitigate the impact of those challenges.

## Conclusions

The ACA is a major change in how the US approaches health care access and financing. Our findings from the early implementation of Medicaid expansion in California offer examples of the kinds of health care reform transition challenges that will need to be addressed by providers and policymakers in the coming years. These include assuring network adequacy in the new ACA-related health plans, addressing financial solvency concerns, improving communication among relevant policymakers and providers, and reducing ACA-related administrative burden. To meet these goals, participants recommended strategies that include maintaining funding for the Ryan White Program, integrating HIV specialty services into new plan networks, increasing collaboration between HIV-specific and non-HIV-specific stakeholders, and hiring support staff to address increased patient enrollment and eligibility needs.
